# Complete Genome Sequences of Six South African Rabies Viruses

**DOI:** 10.1128/genomeA.01085-15

**Published:** 2015-10-01

**Authors:** Claude Sabeta, Baby Phahladira, Denise A. Marston, Emma L. Wise, Richard J. Ellis, Anthony R. Fooks

**Affiliations:** a Agricultural Research Council-Onderstepoort Veterinary Institute, OIE Rabies Reference Laboratory, Pretoria, Republic of South Africa; b University of Pretoria, Veterinary Tropical Diseases Department, Pretoria, Republic of South Africa; c Animal and Plant Health Agency, Weybridge, Surrey, United Kingdom; d Department of Clinical Infection, Microbiology and Immunology, University of Liverpool, Liverpool, United Kingdom

## Abstract

South African rabies viruses (RABVs) from dogs and jackals (canid viruses) are highly related and most likely originated from a single progenitor. RABV is the cause of most global human rabies cases. The complete genome sequences of 3 RABVs from South Africa and Zimbabwe are reported here.

## GENOME ANNOUNCEMENT

Rabies virus (RABV) is the type species of the *Lyssavirus* genus (*Rhabdoviridae* family), which causes a fatal zoonotic disease characterized by encephalomyelitis late in infection. In South Africa, RABV infects many host species. Rabies virus transmission cycles are independently sustained in specific carnivore host species, mainly canine species (*Canidae*), and also mongoose species (principally *Herpestidae*). The domestic dog is the main reservoir and vector species for the transmission of rabies virus in this subregion (similar to other regions in Africa and Asia) (1). Furthermore, in South Africa, in the absence of domestic dogs, RABV (canid biotype) is maintained in wildlife carnivores, such as the black-backed jackal *Canis mesomelas* (2) and bat-eared fox *Otocyon megalotis* (3). The side-striped jackal species, *Canis adustus*, however, cannot maintain RABV independent of disease cycles in dogs (4). Genetic analysis of the rabies viruses from Zimbabwe and South Africa demonstrated a high degree of sequence similarity (≥96.5% sequence identity) between virus isolates originating from dogs and wildlife (jackals), regardless of the enzootic area from which the field specimens were obtained (5, 6). These data emphasize a common progenitor and an ease of exchange of viruses across species barriers. Full-length genome sequences from representative RABVs globally, and in particular from Africa, are essential for understanding these host/viral interactions.

Total viral RNA was extracted from original brain tissues (for South African viruses) and mouse-passaged materials (for Zimbabwean viruses) and prepared for next-generation sequencing (NGS) using the MiSeq platform. Briefly, TRIzol-extracted viral RNA was depleted of host genomic DNA and rRNA, as described previously (7). Double-stranded (ds)-cDNA was synthesized from 50 ng of RNA using a random cDNA synthesis system (Roche), according to the manufacturer’s instructions. The ds-cDNA was purified using AMPure XP magnetic beads (Beckman Coulter), and 1 ng was used for the Nextera XT DNA sample preparation kit (Illumina). A sequencing library was prepared according to the manufacturer’s instructions and sequenced on an Illumina MiSeq with 2 × 150-bp paired-end reads, according to standard Illumina protocols. The total reads were mapped to a reference sequence (accession no. JX473840) using Burrows-Wheeler Aligner (BWA) (v0.7.5a-r405) and were visualized in Tablet, as described previously (7). A modified SAMtools/vcfutils script was used to generate an intermediate consensus sequence in which any indels relative to the original reference sequence were appropriately called. The intermediate consensus was used as the reference for the subsequent iteration of mapping and consensus calling, as described previously (7). The genetic organization of South African canid viral genomes was consistent with that of other previously characterized lyssaviruses (7, 8). In this analysis, the complete genome sequences of 11,923 nucleotides were obtained, with conserved lengths of individual coding regions of the nucleoprotein (N), matrix protein (M), phosphoprotein (P), and the RNA-dependent polymerase (L). Phylogenetic analysis of the sequences revealed geographical grouping. Continued investigations and future analysis of complete RABV genomes will further the understanding of viral evolution and host interaction.

### Nucleotide sequence accession numbers.

The complete genome sequences of the six South African RABVs have been deposited in GenBank under the accession numbers listed in Table 1.

**TABLE 1:** Epidemiological information of the canine viruses sequenced in this study

Virus no.	Lab reference no.	Location of origin	Yr of isolation	No. of reads	% viral reads in brain sample	GenBank accession no.
1	20034	Mutare, Zimbabwe	1991	19,349	0.43	KT336433
2	21057	Muzarabani, Zimbabwe	1992	186,102	1.3	KT336434
3	21467	Goromonzi, Zimbabwe	1993	96,501	2.13	KT336435
4	33512	Gauteng, South Africa	2012	121,722	3.67	KT336436
5	34312	Vhembe, South Africa	2012	11,882	0.31	KT336437
6	55612	North West, South Africa	2012	117,358	4.86	KT336432
